# Thoracic endovascular aortic repair for traumatic and non-traumatic rupture of the descending thoracic aorta: A 15-year single-centre experience

**DOI:** 10.1016/j.ijcha.2025.101818

**Published:** 2025-10-16

**Authors:** Ricarda Berkenheide, Rolf Alexander Jánosi, Fadi Al-Rashid, Daniel Messiha, Konstantinos Tsagakis, Christos Rammos, Sharaf-Eldin Shehada, Payam Akhyari, Thomas Schlosser, Tienush Rassaf, Julia Lortz

**Affiliations:** a Department of Cardiology and Vascular Medicine, West-German Heart and Vascular Center Essen, University of Duisburg-Essen, Essen, Germany. This author takes responsibility for all aspects of the reliability and freedom from bias of the data presented and their discussed interpretation; b Department of Thoracic and Cardiovascular Surgery, West-German Heart and Vascular Center Essen, University of Duisburg-Essen, Essen, Germany. This author takes responsibility for all aspects of the reliability and freedom from bias of the data presented and their discussed interpretation; c Department of Diagnostic and Interventional Radiology and Neuroradiology, University Hospital Essen, University of Duisburg-Essen, Essen, Germany. This author takes responsibility for all aspects of the reliability and freedom from bias of the data presented and their discussed interpretation

**Keywords:** Aorta, thoracic, Aortic rupture, Retrospective studies, TEVAR, Acute aortic syndrome

## Abstract

**Background:**

Ruptures of the descending thoracic aorta are life-threatening emergencies with traumatic and non-traumatic causes. Thoracic endovascular aortic repair (TEVAR) has become a key treatment, but long-term outcome data remain limited. This study aimed to review our experience with TEVAR in patients with traumatic or non-traumatic rupture and identify factors associated with post-TEVAR survival.

**Methods:**

Between 2001 and 2016, 56 patients (21 with traumatic rupture and 35 with non-traumatic rupture) underwent TEVAR at the West-German Heart and Vascular Center Essen, Germany. Data examined included demographics, comorbidities, biomarker levels, imaging results, intervention details, complications, and outcomes (30 days and follow-up).

**Results:**

Patients with non-traumatic rupture were significantly older and had more cardiovascular comorbidities. Patients with traumatic rupture presented more frequently with hemodynamic shock and mediastinal hematoma (47 %). Left subclavian artery coverage was more common in traumatic rupture (57.1 % vs. 18.2 %). Long-term aortic complications were more frequent in non-traumatic rupture (33.3 % vs. 0 %). Patients with traumatic rupture showed significantly longer survival. The overall 30-day mortality was 14.3 % (4.7 % in traumatic rupture patients vs. 20 % in non-traumatic rupture patients) and long-term mortality was 64.5 % (33.3 % in traumatic rupture patients vs. 84.2 % in non-traumatic rupture patients). Age, hypertension, complications, and the aetiology of aortic rupture significantly affected survival.

**Conclusion:**

Patients with traumatic aortic rupture are younger, have healthier vessels, and show better outcomes after TEVAR. This may allow longer follow-up intervals in selected cases, while closer monitoring remains necessary for non-traumatic ruptures.

## 1 Introduction

The rupture of the descending thoracic aorta is a life-threatening event that requires immediate diagnosis and treatment [1]. The underlying aetiology that leads to an aortic rupture is mainly divided into traumatic and non-traumatic reasons. A blunt chest trauma is an example for a traumatic injury of the descending aorta, whereas acute or chronic aortic dissections, intramural hematoma, penetrating aortic ulcer, or an aortic aneurysm might be precursors of a non-traumatic aortic rupture. Direct invasion by adjacent tumours, metastases or iatrogenic aortic injuries are very rare aetiologies leading to non-traumatic aortic injuries.

In recent years, thoracic endovascular aortic repair (TEVAR) has become a first-line therapy for both traumatic and non-traumatic aortic ruptures, now receiving a Class I recommendation in the 2024 European Society of Cardiology (ESC) guidelines [[2], [3], [4], [5], [6], [7]]. While its efficacy in the acute and subacute setting has been demonstrated in numerous studies, and short- to midterm outcomes are well documented [[8], [9], [10], [11]], robust data on long-term outcomes remain limited [12,13]. The purpose of the present study was to analyse the long-term outcome after TEVAR in patients with traumatic and non-traumatic rupture of the descending aorta. Moreover, we aimed to identify differences between patients with traumatic and non-traumatic rupture to derive factors that might affect survival after TEVAR.

## 2 Methods

### 2.1 Patient population

**Fig. 1:**
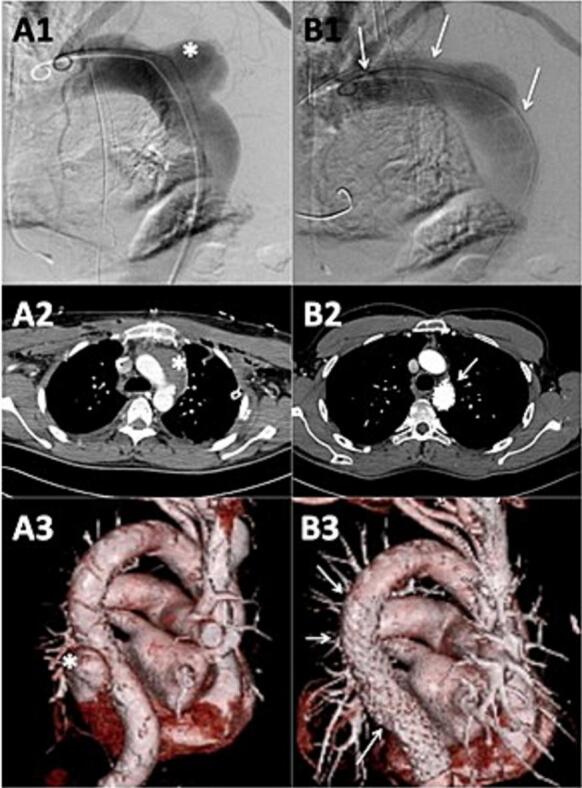
Patient with non-traumatic rupture before (A) and after (B) thoracic endovascular aortic repair. Shown are the pre- and postprocedural results in the angiogram (A1, B1), 2D Computed tomography (CT) −scan (A2, B2) and 3D CT reconstruction (A3, B3). The thoracic rupture spot (*) is treated by an endovascular approach through an aortic stent graft (arrow).

Between 2001 and 2016, 56 patients (40 men, 58 ± 21 years) underwent TEVAR for rupture of the descending thoracic aorta at the West-German Heart and Vascular Center Essen, Germany. Twenty-one patients (15 men, 34 ± 14 years) were treated for traumatic rupture and 35 patients (25 men, 71 ± 9 years) for non-traumatic rupture. Aortic rupture was defined as a disruption of the aortic wall with extravascular collection of blood and/or mediastinal hematoma or hemothorax as documented by computed tomography (CT), transesophageal echocardiography, and/or angiography [14] (Fig. 1).

The study was approved by the local ethics committee of the University of Duisburg-Essen and was conducted in accordance with the principles of the Declaration of Helsinki. Patient records were de-identified and analysed anonymously. The local ethics committee approved the retrospective analysis of patient data without the need to obtain patient consent.

### 2.2 TEVAR procedure

The following stent grafts were used: Valiant and Valiant-Captivia (Medtronic, Minneapolis, MN), Relay respectively Relay Plus (Bolton Medical, Barcelona, Spain), and GORE TAG (W.L. Gore & Ass., Flagstaff, AZ). The stent graft size was either oversized by 10 % (Medtronic, Bolton Medical) or decided based on the range specified by the manufacturer’s guidelines (GORE).

The TEVAR procedure was performed in the cardiac catheterization laboratory by a team of interventional cardiologists, cardiothoracic surgeons, and anaesthesiologists typically under general anaesthesia with mechanical ventilation. However, whenever possible, the procedure was performed under analgosedation without endotracheal intubation in selected cases. The procedure was performed under sterile conditions and intravenous antibiotics (ceftriaxone) were administered prior to its initiation. After surgical or percutaneous exposure of the femoral artery, a standard 6-French arterial sheath was administered and 5000 IU of heparin was injected. Assessment of anatomical conditions, including the location of side branches such as the left subclavian artery and identification of suitable stent graft landing zones, was performed using either digital subtraction angiography with a graduated 6-French pigtail catheter with radiopaque markers and/or intravascular ultrasound (IVUS). Additionally, a 6-French pigtail catheter was advanced from the left radial artery to enable intra-procedural angiography. The stent graft delivery system was advanced over an ultra stiff 0.035 in guide wire (Meier Back-Up; Boston Scientific, Oakland, CA). The stent grafts were positioned in the thoracic aorta under fluoroscopic guidance. Before the stent graft was deployed, systolic blood pressure was lowered to 50 mmHg using intravenous sodium nitroprusside to prevent inadvertent downstream displacement of the stent graft during delivery. Immediate procedural success was evaluated via angiography. No additional heparin or antiplatelet medications were administered following completion of the procedure.

### 2.3 Follow-up

A total of 46 patients survived till discharge, consisting of 20 patients with traumatic rupture and 26 patients with non-traumatic rupture. The patients were integrated into a dedicated outpatient clinic for aortic disease at our institution, ensuring regular clinical visits and imaging follow-up. Five patients were lost to follow-up during the first 12 month. The remaining 41 patients, including 17 patients with traumatic rupture and 24 patients with non-traumatic rupture, underwent a follow-up protocol consisting of clinical examination and imaging of the aorta by contrast-enhanced CT or magnetic resonance imaging (MRI) prior to discharge, after 6 and after 12 months, and annually thereafter, in accordance with the 2014 Guidelines of the ESC [15]. During the first 5 years of observation, 8 additional patients were lost to follow-up, with a further 3 patients lost by 10 years.

### 2.4 Statistical analysis

Continuous variables were presented as mean ± standard deviation or median with minimum–maximum range, while categorical variables were presented using frequencies and percentages. Comparisons were made with the two-sided x^2^ or Fisheŕs exact test for categorical variables and the Mann-Whitney *U* test for metric variables. A p-value of <0.05 was considered statistically significant. The logrank test was used to investigate the effects of the studied parameters on survival. Survival was assessed at 30 days after TEVAR and at the end of the study. The Kaplan-Meier method was used to estimate survival and freedom from aortic reintervention. All statistical analyses were performed using the IBM SPSS Statistics 25 software package (SPSS Inc., Chicago, IL).

## 3 Results

### 3.1 Patient demographics

The baseline characteristics of the patients are provided in Table 1. Compared to patients with traumatic rupture of the descending thoracic aorta, patients with non-traumatic rupture were significantly older and had a greater number of cardiovascular comorbidities, including hypertension, coronary disease, heart failure, and aortic disease. Further, the underlying aortic pathology of patients with non-traumatic rupture was heterogeneous. This included three patients with chronic aortic dissection, two with acute aortic dissection, eleven with penetrating aortic ulcers, thirteen with thoracic aortic aneurysms, and six with two concomitant pathologies, consisting of any combination of the above mentioned conditions, with intramural hematoma being one of the possible additional pathologies. Patients with traumatic rupture showed more frequently hemodynamic instability with hemodynamic shock. Almost half of the patients (47 %) had a mediastinal hematoma.

**Table 1:** Patient baseline characteristics.

	Total (n = 56)	Traumatic (n = 21)	Non-traumatic (n = 35)	p
Age − y	57.5 ± 21.2	34.4 ± 14.4	71.4 ± 8.8	<0.001*
Hypertension	34 (69.4)	5 (26.3)	29 (96.7)	<0.001*
Smoking	11 (26.8)	4 (28.6)	7 (25.9)	0.999
Diabetes	9 (18.0)	1 (5.3)	8 (25.8)	0.127
Obesity	9 (20.0)	4 (23.5)	5 (17.9)	0.711
CAD	17 (32.7)	3 (15)	14 (43.8)	0.032*
PAD	5 (10.0)	1 (5.3)	4 (12.9)	0.637
Previous aortic interventions	10 (18.9)	0 (0.0)	10 (31.3)	0.004*
Impaired LV systolic function	8 (20.0)	0 (0.0)	8 (32)	0.016*
Presence of shock	27 (56.3)	17 (89.5)	10 (34.5)	<0.001*
Hemothorax	28 (54.9)	10 (52.6)	18 (56.3)	0.802
Mediastinal hematoma	13 (26.0)	9 (47.4)	4 (12.9)	0.018*
Positive D-dimer	32 (97)	10 (100)	22 (95.7)	0.999

Data are presented as mean ± standard deviation or n (%). * means significant. CAD, Coronary artery disease; LV, left ventricular; PAD, Peripheral artery disease.

### 3.2 Procedural data

A summary of the procedural data is provided in Table 2. Patients with traumatic rupture were treated at an earlier time frame, with a mean of 17 ± 53 h, whereas patients with non-traumatic rupture were treated more frequently delayed within 74 ± 178 h (p = 0.035). The left subclavian artery coverage was more frequently performed in patients with traumatic rupture (57.1 % vs. 18.2 %, p = 0.003). Three supra-aortic revascularizations were performed in total. One was conducted in a patient with traumatic rupture and a left vertebral artery arising directly from the aortic arch, treated by a hybrid approach with transposition of the left subclavian and left vertebral arteries to the left common carotid artery immediately prior to TEVAR. In addition, one patient underwent a bypass from the left subclavian artery to the ascending aorta, and another received a bypass from the left internal carotid artery to the left subclavian artery. The majority of patients received one stent graft (n = 44, 78.6 %), eight patients (14.3 %) received two stent grafts and three patients (5.4 %) received three stent grafts. Patients with traumatic lesions were mostly treated with one stent graft, but the difference between the two groups was not significant. Residual leakage (type I endoleak) seen during angiography immediately after intervention was observed in four of 56 patients (8.3 %) and required stent graft optimization. One patient with traumatic rupture was treated with repeated balloon dilation, while three patients with non-traumatic rupture were treated with implantation of further stent grafts.

**Table 2:** Procedural data.

	Total (n = 56)	Traumatic (n = 21)	Non-traumatic (n = 35)	p
Interval between diagnosis and intervention − h	53 ± 146	17 ± 53	74 ± 178	0.035*
Stent grafts per patient	1.2 ± 0.6	1.1 ± 0.4	1.3 ± 0.6	0.232
Procedure duration − min	137 ± 72	126 ± 55	146 ± 83	0.628
Volume of contrast agent used − ml	303 ± 182	244 ± 77	341 ± 217	0.247
Coverage of left subclavian artery	18 (33.3)	12 (57.1)	6 (18.2)	0.003*
Transposition	1 (1.8)	1 (4.8)	0 (0)	0.382
Hybrid approach	2 (3.6)	1 (4.8)	1 (2.9)	0.999
Further vascular intervention	4 (7.3)	1 (4.8)	3 (9.7)	0.999
Thoracic drainage	5 (10.6)	0 (0)	5 (17.9)	0.072
Endoleak type 1	4 (8.3)	1 (5)	3 (10.7)	0.631
Periprocedural death	3 (5.4)	1 (4.8)	2 (5.7)	0.999

Data are presented as mean ± standard deviation or n (%). * means significant.

Overall, three patients (5.4 %) died in the periprocedural period. One patient with traumatic aortic rupture died due to the severity of her condition and extensive injuries sustained in a high-impact car accident. The remaining two patients, both with non-traumatic rupture, included one who presented with a perforated thoracic aortic aneurysm larger than 8 cm, which led to complete aortic transection and left no therapeutic options, and another patient who experienced therapy-refractory shock (periprocedurally).

All other patients were successfully treated with TEVAR, and none required conversion to open surgery.

### 3.3 Early outcomes

The significant post-interventional parameters are provided in Table 3. The length of stay at the intensive care unit was 13 ± 15 days (median 7 days), and the length of stay at the hospital was 25 ± 19 days (median 20 days), which showed no significant differences between the two groups. Prolonged ventilation for more than 24 h was required in 22 patients (51.2 %). Acute kidney injury, defined according to the Kidney Disease Improving Global Outcomes (KDIGO) criteria, was the most common complication and developed in 16 patients (34.8 %). This complication mainly occurred in the non-traumatic rupture group (13 vs. 3; p = 0.023).

**Table 3:** Post-interventional parameters.

	Total (n = 53)	Traumatic (n = 20)	Non-traumatic (n = 33)	p
Length of in-hospital stay – d	25 ± 19	30 ± 22	23 ± 16	0.204
Length of ICU stay − d	13 ± 15	14 ± 15	13 ± 16	0.654
Mechanical ventilation > 24 h	22 (51.2)	11 (64.7)	11 (42.3)	0.151
Acute renal injury	16 (34.8)	3 (15.8)	13 (48.1)	0.023*
Renal failure requiring renal replacement therapy	8 (17)	1 (5)	7 (25.9)	0.114
Infection	7 (14.6)	4 (21.1)	3 (10.3)	0.412
Paraplegia	1 (2)	0 (0)	1 (3.2)	0.999
30-day mortality^a^	8 (14.3)	1 (4.7)	7 (20)	0.037*

Data are presented as mean ± standard deviation or n (%). * means significant. ICU, intensive care unit. ^a^ the 30-day mortality is referred to the initial total group size of 56 patients, including the three periprocedural deaths.

In total, eight patients died within the first 30 days (including the 3 periprocedural deaths) resulting in a 30-day mortality rate of 14.3 % (4.7 % in patients with traumatic ruptures and 20 % in those with non-traumatic ruptures; p = 0.037). Of the 7 patients with non-traumatic rupture who died within the first 30 days, 2 had penetrating aortic ulcers and 5 had aortic aneurysms as the underlying pathology.

### 3.4 Long-term outcomes

**Fig. 2:**
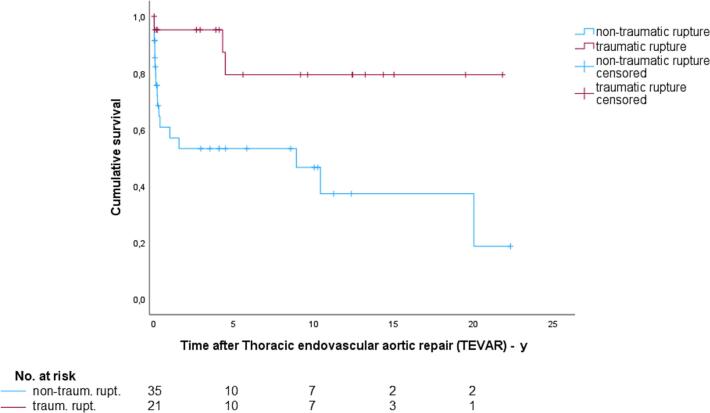
Kaplan-Meier curves for freedom of reintervention (total follow-up) for patients with traumatic and non-traumatic rupture after Thoracic endovascular aortic repair (TEVAR) (Log-Rank test, p = 0.004).

The mean follow-up time was 5.3 ± 6.4 years and patients with traumatic rupture had longer follow-up durations (7.4 ± 6.8 vs. 4.0 ± 5.9 years). During the follow-up period, 8 of 56 patients (14.3 %) required reintervention for endoleaks with or without new rupture, all of whom had non-traumatic ruptures (Fig. 2). The underlying aetiologies for initial TEVAR were penetrating aortic ulcer (n = 4), thoracic aortic aneurysm (n = 3) and acute aortic dissection type B (n = 1). In three patients, the reintervention became necessary during the initial hospital stay. One patient with a type II endoleak within the rupture underwent emergency reintervention but died the following day due to two-staged rupture. Another developed distal stent perforation and died after the procedure from periprocedural myocardial ischemia. A third case involved a pulmonary artery fistula into the stented thoracic aortic aneurysm, which was successfully managed by reintervention. Late reinterventions occured in five patients. These included one patient with a type I and two with type II endoleaks, all with favorable acute outcomes. Another multimorbid patient presented with progressive aneurysm growth proximal to the thoracic stent. Although the reintervention was technically successful, the subsequent hospital stay was prolonged and fatal due to severe pneumonia. In one case of distal stent-induced new entry (dSINE), reintervention was unsuccessful because of severe peripheral arterial disease. However, the patient was stabilized with intensified medical therapy and remained clinically stable during follow-up. A detailed description of reinterventions after aortic rupture is summarized in Table 4.

**Table 4:** Reinterventions after aortic rupture.

Patient No.	Stent graft type	Reason for reint.	Time TEVAR to reint.	Outcome after reintervention
I	Medtronic Talent 38x130 mm	Typ II endoleak within rupture	0.1 months	Emergency reintervention: patient died 1 day after reintervention due to two-stage rupture
II	Medtronic Talent 34x130 mm	Perforation distal stent graft	0.4 months	Patient died 2 days after reintervention due to a periprocedural myocardial ischemia
III	GORE TAG 28x28 mm	Pulmonary artery fistula	0.5 months	Good result after 134.5 months after reintervention
IV	GORE TAG 45x200 mm	Typ II endoleak	1.5 months	Good result after 67 months after reintervention
V	Medtronic Talent 42x100 mm	Typ I endoleak	23.6 months	Good result after 244.5 months after reintervention
VI	Bolton Relay 40x200 mm	Typ I endoleak	61.3 months	Good result after 139.5 months after reintervention
VI	Bolton Relay 32x200 mm	dSINE growth	67.9 months	Unsuccessful reint. due to severe PAD, intensified medical treatment, stable FU after 52.5 months after reintervention
VIII	Bolton Relay 40x60 mm	Progressive aneuryms prox.thoracic stent graft	105.8 months	Patient died 1.3 months after reintervention due to pneumonia

dSINE, distal stent graft-induced new entry; FU, follow-up; PAD, Peripheral artery disease reint., reintervention.

**Fig. 3:**
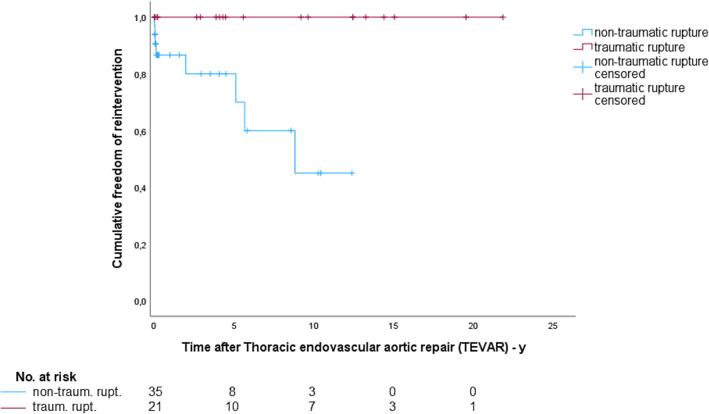
Kaplan-Meier survival curves for patients with traumatic and non-traumatic rupture after Thoracic endovascular aortic repair (TEVAR) (Log-Rank test, p = 0.008).

The overall long-term mortality rate was 64.5 % with better survival observed in patients with traumatic rupture (33.3 % vs. 84.2 %; p = 0.007; Fig. 3).

## 4 Discussion

The use of TEVAR for the treatment of ruptures of the descending aorta represents a key therapeutic approach, particularly for critically ill patients who may not be suitable candidates for open surgery [13,16,17]. Reflecting its proven benefits, the 2024 ESC guidelines have upgraded the recommendation for TEVAR to Class I for the treatment of descending aortic ruptures [5]. This highlights the significant impact of TEVAR on improving survival rates and clinical outcomes in affected patients. Previous studies comparing endovascular and open repair for both traumatic and non-traumatic ruptures have demonstrated that TEVAR was associated with improved survival, fewer complications, including neurological complications such as stroke and paraplegia, and a reduced need for blood transfusions [4,8,18].

In the present study, the overall cohort’s long-term mortality rate was 64.5 % (33.3 % for patients with traumatic rupture and 84.2 % for patients with non-traumatic ruptures), while the 30-day mortality rate was 14.3 % (4.7 % for patients with traumatic ruptures and 20 % for patients with non-traumatic ruptures). One death was observed in the traumatic rupture group, but no further deaths or reinterventions during the hospital stay or the long-term follow-up period were documented. Our results confirm previous findings of improved early survival [10,19], as well as better long-term survival after traumatic rupture [20,21]. Furthermore, none of the patients with traumatic rupture required secondary endovascular intervention during follow-up, which is consistent with earlier studies [20,21]. In contrast, non-traumatic rupture continues to be associated with high mortality [8,18] as well as a frequent need for reintervention [11,13], with our results supporting prior reports [19].

The better outcome observed in patients with traumatic ruptures can be attributed to their significantly younger age, healthier cardiovascular system, fewer cardiovascular risk factors, and not already pre-damaged aortas. In these patients, the aortic tear is typically localized at the aortic isthmus and often can be treated successfully with one stent graft. Although patients with traumatic rupture in this study were more likely to experience hemodynamic impairment and/or mediastinal hematoma, we observed better outcomes in this group after the endovascular procedure, probably due to the factors mentioned above. Because of the typical localization of the rupture at the aortic isthmus, left subclavian artery coverage was also more frequently performed in patients with traumatic rupture.

Nevertheless, the presence of a hemodynamic shock, mediastinal hematoma, or left subclavian artery coverage did not show any association to the outcome after TEVAR in the present study.

The challenge in patients with traumatic aortic rupture is the possible time delay in endovascular or surgical treatment due to other significant injuries, mostly head injury, that has to be taken into consideration [22]. In such cases, an interdisciplinary approach that includes appropriate scheduling of the aortic intervention is critical. TEVAR can be performed before or immediately after other necessary interventions or as a delayed procedure. It is noteworthy that in our study, patients with traumatic rupture were treated sooner than patients with non-traumatic rupture. The reason appears to be twofold: many of the patients with non-traumatic ruptures were referred to our department after a diagnosis at other hospitals. In addition, there were outliers in this group, such as an older patient who agreed to interventional therapy only after 21 days.

Patients with non-traumatic aortic rupture represent a heterogeneous cohort. Non-traumatic ruptures are commonly linked to acute aortic syndrome, including acute aortic dissection, intramural hematoma and penetrating aortic ulcer. In our study, rupture was often triggered by an acute event based on pre-existing aortic disease, such as aortic aneurysms. Patients with non-traumatic rupture were older with significantly higher rates of cardiovascular diseases and risk factors. One-third of these patients had undergone previous endovascular or surgical intervention for the same underlying aortic pathology. The heterogeneity of the underlying aortic pathologies in the non-traumatic rupture group was also reflected in early outcomes. In our cohort, seven patients died within 30 days, including five with ruptured thoracic aortic aneurysms and two with ruptured penetrating aortic ulcers, indicating a particularly high early mortality in patients with aneurysmal rupture. Reported mortality rates from previous studies are in line with our findings, with 30-day mortality rates of around 27–30 % [23,24] for ruptured thoracic aortic aneurysms, 2–18 % [[25], [26], [27], [28]] for acute aortic dissection, and approximately 7 % [29] for complicated penetrating aortic ulcers after TEVAR. Non-traumatic aortic rupture patients have significantly higher rates of aortic complications and reinterventions in the early and late follow-up periods and, therefore, regular follow-up intervals are of the utmost importance. CT or MRI of the aorta are recommended at 1, 6, and 12 months, and yearly thereafter, according to the guidelines on the diagnosis and treatment of aortic diseases from the ESC from 2014. Imaging should also be employed in the case of new or renewed symptoms [11]. In 2024, the new ESC Guidelines for the management of peripheral arterial and aortic diseases were published, recommending post-TEVAR CT imaging at 1,6 and 12 months, followed by annual scans for at least the first 5 years after the intervention, then less frequently if no complications are detected, in patients treated with TEVAR for acute aortic syndrome [5].

Especially young patients with traumatic ruptures need consequent follow-up due to their high life expectancies, since data regarding the long-term durability of stent grafts are rare. Long-term follow-up examinations are inevitable, not only because of the aortic dilatation distal or proximal of the stent graft. Further studies are needed, since the clinical impact is still unknown [20]. At the same time, repeated CT imaging over decades may pose a considerable cumulative radiation burden for these often young patients. Therefore, the Society for Vascular Surgery has suggested that in patients with blunt traumatic aortic injury (BTAI) treated by TEVAR, follow-up intervals may be extended to every two to five years when no abnormalities are present [30]. Similarly, a recent *meta*-analysis of TEVAR after BTAI proposed that imaging may be focused on the first year and at five years in otherwise healthy young patients, as reinterventions were rare and typically occurred at these time points [31].

## 5 Limitations

The present study is a single-centre retrospective study over a period of 15 years. Because of the study’s retrospective design, some data were incomplete and the sample sizes of various parameters were different. Furthermore, the study was based on a small number of patients and further multi-centre or prospective studies are needed to confirm its results. In addition, due to the long observation period ending in 2016, it must be considered that methods, materials, and also the expertise of the interventionalists have evolved over time, which may have influenced the outcomes. Moreover, the non-traumatic rupture group was heterogeneous. Given the small size of these subgroups, potential differences in outcomes may not be fully captured and the overall mortality reported for the group may not accurately reflect the risk associated with each specific pathology.

## 6 Conclusion

TEVAR is an effective treatment for both traumatic and non-traumatic ruptures of the descending thoracic aorta, with significantly better outcomes observed in younger patients with traumatic ruptures. These patients had lower mortality, fewer reinterventions, and longer survival compared to those with non-traumatic ruptures. Differentiating long-term follow-up schedules according to rupture type appears reasonable. In patients with traumatic rupture who remain stable during the first 24 months, extending the interval between imaging studies to one to two years may be appropriate. Further research is needed to assess the long-term durability of stent grafts, particularly in younger patients with traumatic ruptures.

## CRediT authorship contribution statement

**Ricarda Berkenheide:** Writing – review & editing, Writing – original draft, Visualization, Validation, Project administration, Methodology, Formal analysis, Data curation, Conceptualization. **Rolf Alexander Jánosi:** Writing – review & editing, Supervision, Methodology, Investigation, Formal analysis, Data curation, Conceptualization. **Fadi Al-Rashid:** Writing – review & editing, Investigation, Data curation, Conceptualization. **Daniel Messiha:** Writing – review & editing, Methodology, Data curation, Conceptualization. **Konstantinos Tsagakis:** Writing – review & editing, Visualization, Project administration, Methodology, Investigation, Formal analysis, Data curation, Conceptualization. **Christos Rammos:** Writing – review & editing, Visualization, Project administration, Investigation, Data curation, Conceptualization. **Sharaf-Eldin Shehada:** Writing – review & editing, Visualization, Investigation, Conceptualization. **Payam Akhyari:** Writing – review & editing, Validation, Supervision, Project administration, Conceptualization. **Thomas Schlosser:** Writing – review & editing, Visualization, Validation, Software, Methodology, Formal analysis, Data curation. **Tienush Rassaf:** Writing – review & editing, Project administration, Methodology, Investigation, Formal analysis, Data curation, Conceptualization. **Julia Lortz:** Writing – review & editing, Writing – original draft, Visualization, Validation, Supervision, Project administration, Methodology, Formal analysis, Data curation, Conceptualization.

## Funding

This research did not receive any specific grant from funding agencies in the public, commercial, or not-for-profit sectors.

## Declaration of competing interest

The authors declare that they have no known competing financial interests or personal relationships that could have appeared to influence the work reported in this paper.
